# Real-Time Multi-Channel Epileptic Seizure Detection Exploiting an Ultra-Low-Complexity Algorithm–Hardware Co-Design Approach

**DOI:** 10.3390/s25226889

**Published:** 2025-11-11

**Authors:** Andrea Vittimberga, Giovanni Nicolini, Giuseppe Scotti

**Affiliations:** Department of Information, Electronics and Telecommunication Engineering, Sapienza University of Rome, 00184 Roma, Italy; andrea.vittimberga@uniroma1.it (A.V.); giovanni.nicolini@uniroma1.it (G.N.)

**Keywords:** electroencephalogram, intracranial EEG, adaptive neurostimulation, epilepsy, seizure onset detection, FPGA

## Abstract

This paper presents an automated threshold-based multi-channel epileptic seizure detection algorithm designed for low-complexity hardware implementations. The algorithm relies on two discriminative, computationally simple time-domain features, based on power and amplitude variations, that enable accurate and timely detections due to their rapid adaptiveness to fluctuations in neural activity. To ensure long-term functionality and high sensitivity, system thresholds are optimized through an offline calibration process that exploits the statistical analysis of patient-specific inter-ictal and ictal periods. The novelty of the approach lies in its multi-channel decision-making strategy, which enhances reliability against false alarms. The proposed algorithm is tested on multiple datasets to assess its adaptability to different recording conditions, achieving roughly 98% accuracy and over 98% sensitivity on both the EEG CHB-MIT dataset and the iEEG SWEC-ETHZ dataset, with average latencies of 3.37 s and 7.84 s, respectively. These results are comparable to, and in some cases outperform, several published machine-learning-based approaches. On the hardware side, FPGA synthesis highlights the minimal and scalable resource requirements of the proposed architecture, achieved through Time-Division Multiplexing (TDM) of both filtering and feature extraction. When compared to state-of-the-art proposals, the system emerges as an ideal candidate for real-time, resource-constrained hardware implementations.

## 1. Introduction

Epilepsy remains one of the most prevalent neurological disorders worldwide, and while pharmacological treatments are effective in many cases, approximately a quarter of the affected individuals fail to respond adequately to conventional medications [1,2]. As a consequence, recent advancements have focused on the development of automated device-based approaches to address the challenge of refractory epilepsy. Among these, techniques like Vagus Nerve Stimulation (VNS) and Deep Brain Stimulation (DBS) rely on the localized delivery of electrical stimuli to ameliorate the effects of the ongoing seizure in a controlled manner [3,4,5].

In its conventional, open-loop form, DBS delivers continuous electrical pulses to targeted brain regions according to pre-set parameters, without taking into account the patient’s current neural state. Adaptive variations in DBS, however, enact neuromodulation based on real-time monitoring of the subject’s neural responses, dynamically adjusting stimulation to optimize therapeutic outcomes and reduce side effects [6,7]. For this reason, automated seizure detection is becoming increasingly prevalent as an ideal candidate to regulate the stimulation protocol.

Traditional seizure detection has typically relied on visual inspection of neural recordings, a process that is inherently subjective, time-consuming, and therefore ill-suited not only for automated detection but also for deployment in implantable devices. Developing objective, efficient, and reliable methods for automated seizure detection presents a critical hurdle due to the strong variability of pre-ictal and inter-ictal patterns both within individual patients and across different patients, as well as the non-stationary nature of scalp EEG and intracranial EEG (iEEG) signals. This high variability complicates the design of generalized detection algorithms and introduces an inherent trade-off between sensitivity and accuracy, as systems must balance the minimization of both false positives and false negatives. These challenges make it difficult to develop algorithms capable of maintaining robust performance across diverse individuals and recording conditions.

A key task in seizure detection involves identifying optimal representations from neural signals within the feature space. From this perspective, a plethora of signal descriptors and metrics has been proposed and evaluated in terms of their discriminative capabilities. In this context, features may be extracted from the time domain, including peak density, signal power, or Hjorth parameters [8,9]. Furthermore, non-linear features like Approximate Entropy (ApEn) have seen increased usage, adding complementary information to traditional time-based metrics [10]. In frequency analysis, Cross-Frequency Coupling (CFC) stands out in particular, with Phase-Amplitude Coupling (PAC) being widely employed to quantify the relationship between the amplitude of high-frequency oscillations and the phase of slower frequency bands [11].

More recently, machine-learning and deep learning strategies have taken center stage: models such as Support Vector Machines (SVM), Convolutional Neural Networks (CNN), and other architectures are able to combine and process large sets of features, achieving high levels of accuracy in seizure onset detection and prediction [12,13]. Despite their strong performance, these methods often involve considerable computational overhead, which limits their practical use in implantable systems. Such devices must function under strict constraints on area and power consumption, conditions that remain difficult to meet with current state-of-the-art algorithms. The study in [14] proposes a patient-specific, FPGA-implemented, threshold-based detection algorithm that leverages eleven time-domain features. A feature ranking strategy is employed to identify the most discriminative subset of features, while a channel selection mechanism is integrated to reduce data dimensionality. However, the initial number of extracted features is relatively high, and the resources required for their computation, as well as for the associated pre-processing steps, are non-trivial. On that topic, the work presented in [15] aims to reduce the computational load by restricting the set of features to a single time-domain Local Binary Pattern (LBP) code extracted from the recorded iEEG signal. While effective, the algorithm has not been implemented on hardware platforms and presents significant latency. Additionally, the work proposed in [16] employs a combination of under-sampling and boosting techniques to improve the performance of a classifier, which in turn makes use of statistical metrics extracted from pre-processed EEG signals. Although the original paper does not report implementation details, it can be reasonably assumed that calculating higher-order metrics, such as kurtosis or skewness, could limit the applicability of the approach on portable or resource-constrained hardware. In [17], the authors proposed an adaptive distance-based seizure detector which makes use of both statistical and morphological metrics to elaborate signals that undergo principal component analysis and common spatial patterns to enhance the available data. Similar potential limitations as [16] also apply in this case.

Regarding more specific implementations that target hardware realization on FPGA, the system proposed in [18] implements a mixed-signal SoC featuring an analog front-end for EEG acquisition and a digital detection core in 55 nm CMOS technology. The detection unit performs on-chip feature extraction based on four eigenvalues derived from time-domain features. Hardware modules handle feature calculation, threshold update, and detection logic. The design was prototyped on FPGA and later realized as an ASIC.

In addition, the system presented in [19], implemented on an Artix-7 FPGA, realizes energy-efficient, real-time seizure detection. The hardware consists of dedicated modules for feature extraction, calculation of Hjorth mobility and non-linear energy in parallel pipelines, and on-chip SVM or QDA classifiers. The design includes optimized memory buffers and control logic to manage data flow between extraction and classification units, achieving minimal dynamic power (approximately 0.057 mW for SVM). In both of the aforementioned cases, there remains margin for improvement in terms of resource allocation.

In this work, we propose a novel framework to analyze and detect abnormal patterns related to epileptic seizure events, based on the power fluctuations of the EEG signal, and applied through the use of two discriminative time-domain features of simple derivation. The proposed algorithm is threshold-based and, as such, it is much simpler in terms of hardware resources with respect to the aforementioned solutions, yet it is able to achieve state-of-the-art comparable performance regarding accuracy, sensitivity, and latency. The novelty of this work lies in the combination of a minimal feature set, consisting of Line Length (*LL*) and instantaneous power difference (*PD*) between consecutive samples, which enables a simple and low-complexity algorithm. The *PD* feature benefits from being compatible with partial LL computation, as both require only two samples at a time, reducing hardware requirements.

In addition, instead of implementing a channel reduction approach, a full-channel majority-voting mechanism leverages all recording channels to improve true positive detection while limiting false alarms, made possible by the low-complexity algorithm and minimal hardware usage. Finally, a hardware-oriented Time-Division Multiplexing (TDM) architecture executes filtering and partial feature extraction within the TDM framework, allowing scalable operation across a large number of channels and making the system suitable for implantable applications.

The paper is organized as follows: Section 2 is devoted to the presentation of the proposed work, from the employed set of features to the detailed description of the algorithm’s functionality in its distinct steps, including the pre-processing stage. Section 3 delves into the details of the hardware implementation of the proposed seizure detector, showing its functionality with simulations conducted across different software environments. Section 4 reports the simulation results obtained using EEG and iEEG datasets as benchmarks for system validation, providing an extensive description of the statistical metrics employed to assess the algorithm and tune its parameters. In addition, this section hosts a comparative analysis that weighs the algorithm’s performance and synthesis on FPGA against current state-of-the-art proposals. Finally, Section 5 concludes the work.

## 2. Proposed Work

This section details the main components of the proposed seizure detection system, starting with a description of the double feature extraction stage that provides the basis for the algorithm. Consequently, the multi-channel signal processing flow and threshold-based decision rule used to detect the seizure onset are thoroughly explained.

### 2.1. Features

Feature extraction is a critical step in seizure detection algorithms, as it transforms the filtered neural signals into representations that highlight the more relevant patterns pertaining to the ictal activity. In this work, two time-domain features were selected based on their discriminative capability, computational efficiency, and ability to follow variations associated with seizure onsets with relatively low latency. In limiting the feature set to two, the overall complexity of the extraction stage is greatly reduced, making the system suitable for resource-constrained hardware implementations. The specific features to be extracted in the proposed algorithm are introduced and discussed in the following.

#### 2.1.1. Power Difference

As seizure events occur, they manifest as sudden, abnormal bursts of synchronized neural activity, often observable through sharp increases in amplitude and shifts in frequency. To characterize rapid changes in signal dynamics, the first of the two considered features quantifies the variation in instantaneous power by computing the difference between the squared values of consecutive samples. Such power difference (*PD*) can be analytically expressed as

(1) $$PD=x{\left[n\right]}^{2}-x{\left[n-1\right]}^{2}$$

where x[n] denotes the amplitude of the *n*-th sample. The *PD* feature is effective in capturing instantaneous power and frequency fluctuations with reliable consistency, as it emphasizes localized shifts in amplitude, allowing it to distinguish between pre-ictal and ictal states.

#### 2.1.2. Line Length

Line length is a time-domain feature widely used in EEG/iEEG signal analysis due to its high sensitivity to variations in both amplitude and frequency [20,21]. It is computed as the cumulative sum of the absolute differences between consecutive signal samples within a given time window of *N* samples, as defined in [22]:

(2) $$LL=\sum_{n=1}^{N}\left|x[n]-x[n-1]\right|$$

In the initial stages of the ictal state, neural signals often exhibit subtle changes, such as low-amplitude rhythmic oscillations or minor waveform distortions. These early alterations may not be efficiently captured by the power difference feature alone, which, being quadratic in nature, responds more prominently to abrupt, localized increases in amplitude while tending to suppress smaller fluctuations, potentially resulting in delayed seizure onset detection. In contrast, the line length feature offers greater sensitivity to these early dynamics and, through its window-based cumulative process, generally yields a smoother, monotonic increase in value that reflects the progression of the seizure event.

### 2.2. Proposed Algorithm

**Figure 1:**
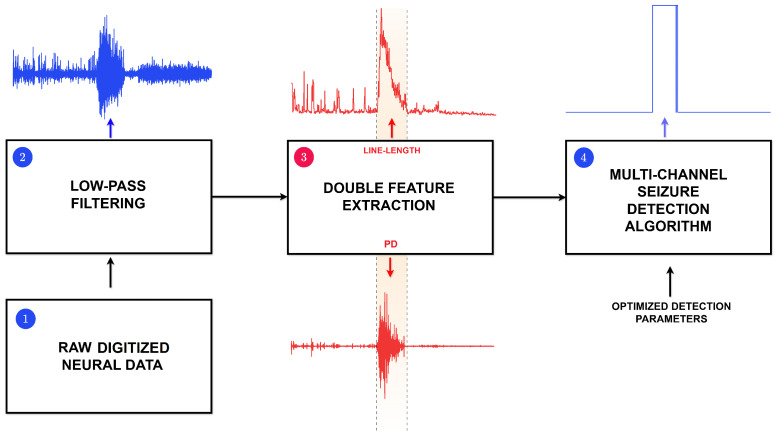
General diagram of the proposed algorithm: (1) neural data are digitized by the analog front-end, and (2) low-pass filtered for pre-processing. (3) Main time-domain features are extracted, and (4) aggregated information is processed to detect seizure-related activity.

The proposed seizure detection algorithm, which is outlined in Figure 1, is based on a thresholding principle that fully harnesses information provided by multi-channel neural recordings to achieve a consistent and responsive discrimination. Particularly, since seizure events are typically characterized by pronounced fluctuations, the proposed algorithm leverages the *LL* and *PD* features to track and locally enhance such variations with respect to inter-ictal neural dynamics. The combination of these two extracted features raises the probability of correctly distinguishing false alarms from true epileptic events. To that end, an initial offline calibration stage is employed to determine the optimal detection thresholds, thereby accounting for patient-specific variability and recording conditions, as well as ensuring that the algorithm is able to operate reliably in a real-time scenario.

A window-based processing scheme is used to improve the temporal resolution of detection and to organize the processing pipeline in a structured, stepwise manner. With an appropriately chosen window length *N*, this approach also enhances the consistency of the analysis. Indeed, windows that are too large can reduce the algorithm’s discriminative ability and cause increases in latency, whereas excessively short windows may fail to capture the characteristic fluctuations between ictal and inter-ictal states or make the system more susceptible to false detections. For each window, the algorithm first extracts the *PD* and *LL* features after low-pass filtering of the signal is performed. Next, seizure discrimination is carried out by monitoring whether these features cross the detection thresholds. The remainder of the section presents a detailed description of the general processing chain involved in the proposed methodology.

#### 2.2.1. Data Pre-Processing

Pre-processing is deliberately kept to a minimum in order to maintain a lightweight algorithm that is well-suited for resource-constrained applications. In particular, advanced artifact-removal techniques such as Independent Component Analysis (ICA) are not employed, as they introduce additional computational complexity and risk discarding signal components that may still contain relevant seizure-related information.

**Figure 2:**
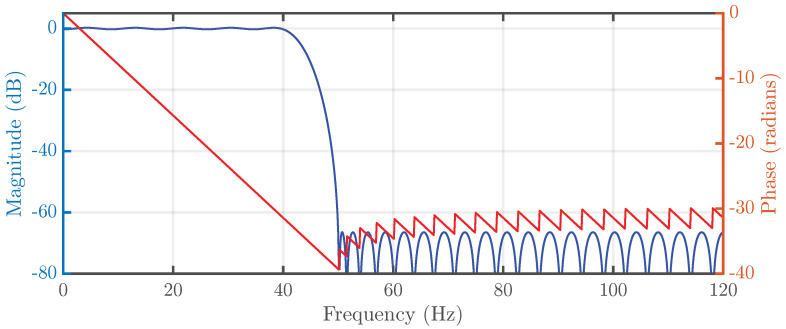
64-taps FIR filter magnitude and phase response.

A 64th-order low-pass Finite Impulse Response (FIR) filter, implemented using TDM across recording channels, is applied to the incoming neural data. The FIR structure ensures a linear phase response, which preserves the temporal coherence of the EEG signals. The stop-band frequency is set to 50 Hz (Figure 2), effectively suppressing high-frequency artifacts and noise while retaining the spectral bands most relevant for seizure detection according to our study, namely Delta, Theta, Alpha, and Beta. Furthermore, the selected order guarantees an attenuation of at least 60 dB at 50 Hz, ensuring robust rejection of power-line interference.

This filter is included in the processing chain regardless of any pre-processing already applied to the test datasets, providing a complete system that can operate directly on in vivo neural data and remain adaptable for real-time implementation.

#### 2.2.2. Seizure Detection Algorithm

**Figure 3:**
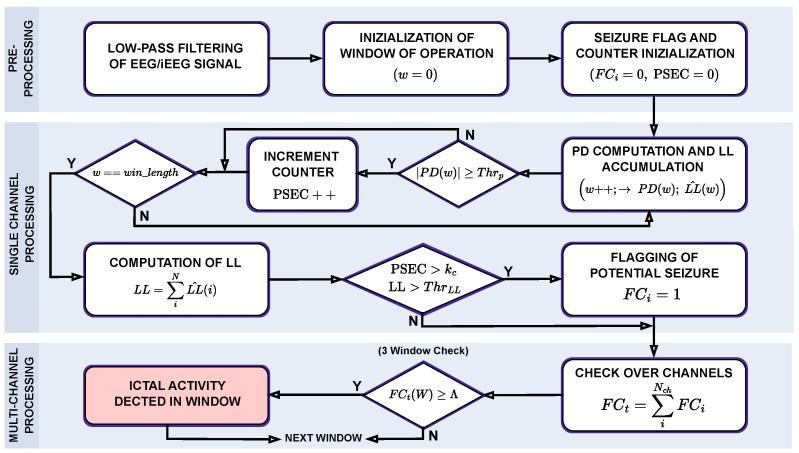
Pseudo-algorithmic flow-diagram of the proposed seizure detection algorithm. The steps are grouped by their functionality within the processing chain.

The overall algorithm is summarized in the high-level flow diagram shown in Figure 3. Its operation can be divided into two main stages: single-channel processing and multi-channel decision-making. The first stage focuses on the analysis of each EEG (or iEEG) channel independently, while the second stage integrates information across multiple channels to provide a final decision on seizure occurrence.

As a preliminary step, the acquired neural signals undergo low-pass filtering to suppress high-frequency noise and reduce the influence of potential artifacts, as described in Section 2.2.1. Following this pre-processing step, the algorithm proceeds in a window-based fashion. At the beginning of the analysis, corresponding to the first sample of the first window, all relevant parameters are initialized to zero. Specifically, the window sample-related variable *w* is set to 0, and for each recording channel two dedicated variables are initialized: (i) the Potential Seizure Event Counter (PSEC), which tracks the number of *PD* threshold crossings within the window under analysis for a given channel, and (ii) the Flag Counter (FC), which is later used in the decision stage to identify the presence of epileptic seizure evidence shared among channels.

At this point, the single-channel processing phase is triggered. In particular, following a first increment of the sample variable *w*, the *PD* is computed according to Equation (1). Meanwhile, the algorithm updates the running accumulation of the *LL* feature, with the partial value being defined as $\hat{\mathrm{LL}}\left(w\right)$. Since *LL* is defined as an accumulated measure, only this intermediate value is provided during window progression, whereas the final value of the accumulation, given in Equation (2), becomes available only at the end of the window under analysis.

The first condition in the detection process evaluates the absolute value of the extracted *PD* at each sample against a predefined threshold, $Thr_{p}$. Such threshold is obtained from an offline statistical analysis of a sufficiently large dataset to ensure proper tuning, accounting for the trade-off between detection sensitivity, latency, and overall accuracy. Whenever the threshold is exceeded, it indicates a potential seizure event for the corresponding channel, resulting in an increment of PSEC.

(3) $$\mathrm{PSEC}=\left\{\begin{array}{cc} \mathrm{PSEC}+1, & \mathrm{if}\;\left|PD(w)\right|\geq Thr_{p},\\ \mathrm{PSEC}, & \mathrm{otherwise}. \end{array}\right.$$

This process is reiterated for each sample in the current analysis window until all samples have been processed. Once the last sample is evaluated, the window is complete, and the algorithm can proceed to the next state of the flow diagram. Here, the *LL* is available to be used for further evaluations. In particular, a double condition is checked in order to potentially raise the seizure flag FC, and it is defined as follows:

(4) $$\left\{\begin{array}{c} \mathrm{PSEC}\geq k_{c},\\ LL\geq Thr_{LL} \end{array}\right.$$

where $k_{c}$ represents an empirical factor that quantifies the peak density of the *PD* feature with respect to its threshold within the analysis window, while $Thr_{LL}$ denotes the threshold applied to the *LL* accumulated over the same window. Both parameters, similar to $Thr_{p}$, are determined offline through a calibration process aimed at optimizing system performance. The system of equations in Equation (4) implements a logical OR operation, meaning that if either condition is satisfied the samples within the current window are flagged as abnormal, and the flag counter $FC_{i}$ is set to 1, where *i* denotes the *i*-th channel.

Although the use of a logical OR operation makes the detection framework inherently more permissive compared to a stricter AND condition, thereby potentially increasing vulnerability to false positives, the actual impact on the False Positive Rate (FPR) remains limited when the calibration process is properly carried out. In practice, the thresholds are tuned in a way that balances the contribution of the *PD* and *LL* features, allowing them to complement one another. This compensation mechanism ensures that the system can achieve an improved trade-off between sensitivity and latency without a significant degradation in accuracy.

The final stage of the algorithm integrates a multi-channel voting strategy to determine the effective presence of ictal activity. Specifically, the seizure flags from all channels are aggregated, and the percentage of marked channels is compared against a predefined threshold, denoted as Λ. A seizure is declared only if the number of flagged channels exceeds Λ, thus enhancing robustness against spurious detections on individual channels:

(5) $$FC_{t}=\sum_{i}^{N_{ch}}FC_{i}\geq \Lambda$$

with $FC_{t}$ being the sum of the flags $FC_{i}$ across all processing channels. To further improve the system’s reliability, the condition presented in Equation (5) is required to be satisfied over three consecutive windows, ensuring that the detected activity corresponds to a sustained ictal pattern rather than a non-ictal fluctuation.

## 3. Hardware Implementation

The following section provides a detailed account of the hardware implementation of the various functional blocks that compose the digital processing chain underlying the proposed epilepsy detection algorithm. In that regard, the FPGA platform AMD Artix-7 was considered for the design’s realization. Specifically, the proposed processing chain was implemented as a hardware-oriented simulation model in Simulink, using the AMD proprietary System Generator libraries, integrated within the Vitis Model Composer tool.

This methodology allowed the proposed system to be thoroughly tested and validated within a controlled software environment, while simultaneously supporting the automatic generation of synthesizable HDL code. As such, the resulting system was simulated both within the Simulink environment and the Vivado Design Suite using the CHB-MIT scalp EEG dataset [23], which will be described in more detail in Section 4.1.

### 3.1. FIR Filter

As previously mentioned, the processing chain includes a 64th-order low-pass FIR filter, implemented using a TDM approach. The choice of a FIR structure over an Infinite Impulse Response (IIR) alternative ensures a linear phase response, thereby preserving the temporal integrity of the EEG signals and avoiding distortions that could compromise feature extraction. To optimize resources usage, the TDM approach enables the reuse of a single DSP unit across the multiple input channels, which significantly reduces hardware complexity while still meeting the required throughput.

**Figure 4:**
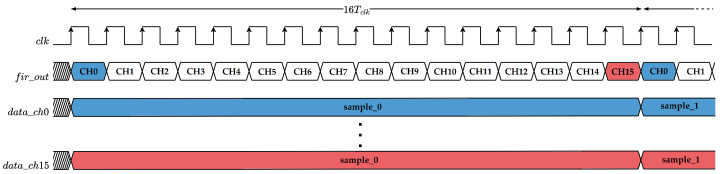
Timing diagram of the FIR filter operation, illustrating the sequential time-division multiplexing process for a generic case with 16 channels.

The filter is designed to operate at a sufficiently high clock frequency to handle all available recording channels in real time. Specifically, the operating frequency is defined as

(6) $$f_{FIR}\geq f_{s}\cdot N_{ch},$$

where $f_{s}$ is the input sampling frequency, and $N_{ch}$ represents the number of channels. This relation ensures that the filter can sequentially process the *i*-th sample of each channel across all coefficients before the next input sample arrives. The high-level functional timing diagram of the FIR’s operation is shown in Figure 4. For illustrative purposes, the diagram refers to a simplified case study with 16 input channels.

**Figure 5:**
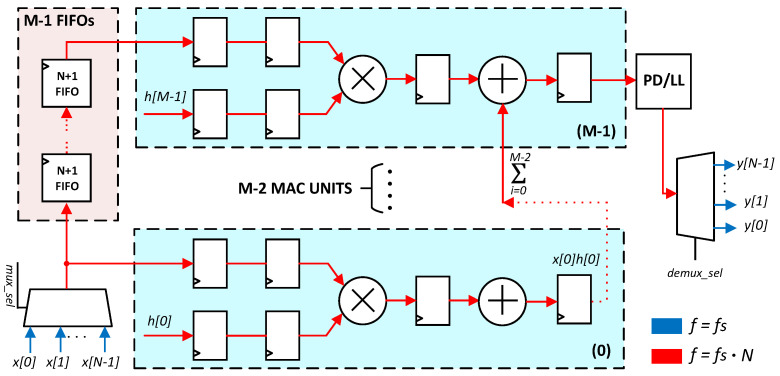
DSP slice of a multi-channel FIR filter operating in TDM.

The filter coefficients were generated with MATLAB’s Filter Design and Analysis (FDA) tool and provided as input to the Vivado FIR Compiler, which handles coefficient quantization and hardware mapping. In this implementation, the coefficients are represented with a 16-bit word length, which ensures that the realized frequency response closely matches the ideal design. The underlying architecture adopted for the FIR is a systolic Multiply-Accumulate (MAC) structure, well suited for FPGA-based realizations (Figure 5). In this architecture, data flows through an array of interconnected processing elements, each performing a partial multiplication and accumulation. This pipelined organization improves resource utilization, allowing the filter to sustain real-time operation across multiple channels while maintaining scalability and predictable timing behavior. For data alignment purposes, a series of First-In First-Out (FIFO) elements is used to ensure proper TDM operation, while dedicated registers are inserted in the processing chain to break critical paths.

To further optimize resources usage, part of the FIR FIFO used to delay the input data is implemented using Block RAM (BRAM) elements instead of flip-flops, thereby reducing the overall register count and improving area efficiency on the FPGA.

### 3.2. Double Feature Extractor

**Figure 6:**
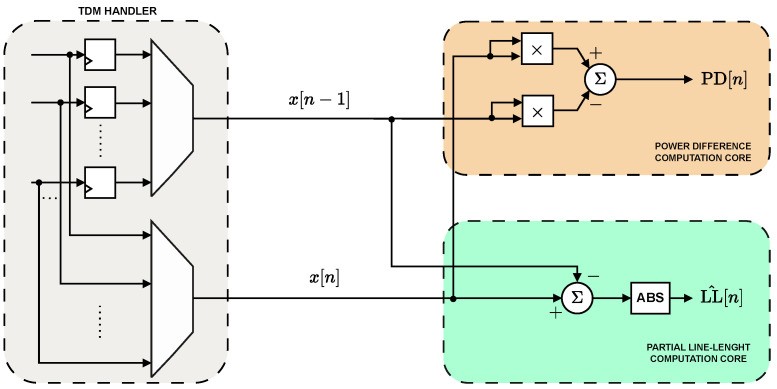
Hardware architecture of the TDM double feature extractor.

The hardware implementation of the double feature extracting core benefits from the TDM architecture as well, specifically with regard to resource minimization (Figure 6). The input data stream coming from the FIR filter is first demultiplexed, to account for the sequential logic required by the definition of the *PD* and *LL* feature, and then multiplexed again to perform the TDM operation. This approach enables the reuse of combinational logic across channels, significantly reducing hardware overhead. Consequently, the *PD* feature is implemented in TDM using two multipliers and a single subtractor.

The same multiplexed processing is applied to the computation of the absolute difference between consecutive samples required for the *LL* feature. However, unlike the *PD*, the accumulation over the full window cannot be shared across channels, as each channel requires an independent sum of its differences. For this reason, the *LL* implementation employs a dedicated accumulator per channel, while keeping the absolute-difference computation time-shared across the multiplexed data stream.

### 3.3. Detection Unit

After filtering and processing by the TDM double feature extractor, the data stream is sent to a demultiplexer and, subsequently, to the detection units, which include counters, comparators, and a deglitch module apiece. Each *PD* sample triggers a comparator when the condition in Equation (3) is satisfied, enabling an accumulator that increments by one unit and is reset at the end of the operational window through dedicated logic. A second comparator evaluates the peak density within the window, while, in parallel, a third one assesses the *LL* at the end of the same temporal interval. The outputs of the two comparators are combined using a logical OR and drive specific logic that triggers a flag whenever either the PSEC or the accumulated line length exceeds their respective thresholds.

A dedicated deglitch unit, which is a temporal filter, stabilizes the output over a defined time interval, with its operation being controlled by an edge detector that monitors the comparators’ outputs.

Regarding the multi-channel voting, after proper type conversion, the single detectors’ outputs are fed to a tree of adders, which sum the contributions from each channel to determine the percentage that have flagged potential ictal activity. The output of the summing operation is evaluated by a comparator against the threshold Λ, implementing the condition described in Equation (5). Once again, a deglitch unit is exploited to stabilize the comparator’s output, which is subject to a final check by dedicated logic over three consecutive windows.

### 3.4. Architecture-Level Hardware Simulation

**Figure 7:**
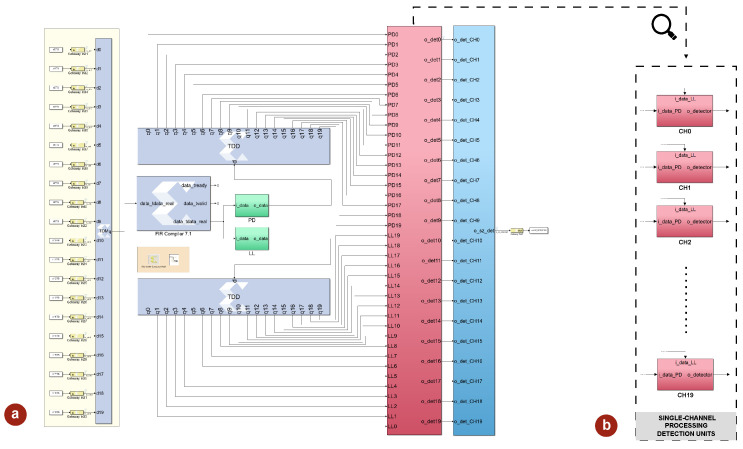
(**a**) Simulink model of a 20 channel implementation of the proposed algorithm. (**b**) Focus on the single-channel detection blocks that comprise the overall detection unit.

Shown in Figure 7a is the model of the hardware architecture implementing a 20-channel version of the proposed algorithm, emphasizing the scalability of the system. The choice of twenty channels was deemed sufficient to meaningfully exploit the multi-channel voting mechanism, while still maintaining a realistic hardware configuration. As shown at the top of the figure, the 20 EEG inputs logged from the MATLAB environment are collected and passed through a dedicated time-multiplexing block, which combines them into a single interleaved stream. This stream, operating at the frequency specified in Equation (6), is directed to the FIR filter stage, whose output signal is then delivered to the dual feature-extraction unit, responsible for producing the *PD* and partial *LL*. After said extraction, the TDM streams are demultiplexed to reconstruct 20 parallel signals per feature at frequency $f_{s}$, which are finally routed to the individual detection units, where the initial stage of the decision process is carried out independently on each channel (Figure 7b).

**Figure 8:**
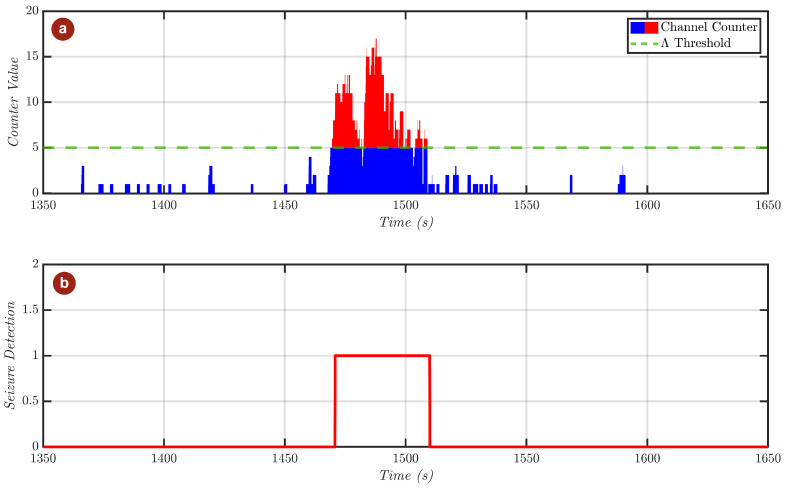
Detector’s output simulated in Simulink. Depicted are (**a**) the output of the channel voting unit, along with (**b**) the actual seizure detection result.

The final element of the system incorporates the multi-channel voting block that was previously described in detail. To demonstrate the proper functionality of the system at hardware-level, the output of the detector when fed with a seizure-affected sample is presented in Figure 8.

Notably, it can be observed that, as the ictal state begins, the number of channels that flag the presence of seizure-related activity (Figure 8a) starts incrementing rapidly, as a consequence of both the increased density of *PD* peaks and the elevated *LL* values observed across multiple channels. The multi-channel voting mechanism continuously monitors this count, and once the latter exceeds the predefined threshold Λ, set to 5 in this example, for at least three consecutive windows, the detector output is activated, as illustrated in Figure 8b.

**Figure 9:**
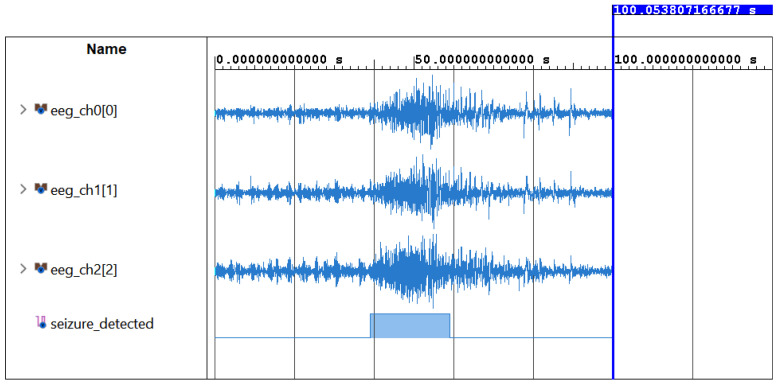
Vivado simulation reporting key waveforms of the algorithm’s RTL implementation. Depicted are the three input EEG data taken from the CHB dataset, and the output of the detector.

The system was then imported into the Vivado Suite to be synthesized and to perform a functional simulation. Figure 9 shows the result of the detection when fed with EEG data capturing a segment that includes the transition from non-ictal to ictal activity. For visual clarity, only three channels are present in the waveform window. In this case, it is evident that the system reacts rapidly to the presence of abnormal seizure-related patterns.

### 3.5. FPGA Synthesis Results

To validate the simplicity of the proposed work, the overall architecture was synthesized on an FPGA platform, namely the AMD Artix-7 XC7A100T FPGA. Without loss of generality, a 4-channel version was prepared for the sake of the synthesis, in order to be reasonably comparable with state-of-the-art single-channel implementations while keeping the multi-channel facet peculiar to the proposed algorithm. The resulting report is compiled in Table 1, showing the exact unit utilization, correlated with the percentage of the same with respect to the available resources on the chosen platform. As can be observed, the reported results corroborate the low complexity and efficiency of the proposed architecture, confirming its suitability for real-time implementation on resource-constrained hardware.

## 4. Experimental Results and Comparative Analysis

This section describes the simulation framework, with a focus on the datasets used for tuning and validating the algorithm in the MATLAB 2021a environment. Key figures of merit employed to assess the detector are also presented. Finally, the results of applying the algorithm to the CHB-MIT and SWEC-ETHZ datasets are reported and analyzed in detail and compared against current state-of-the-art proposals. Additional comparisons will also address the hardware resource allocation with respect to other implemented designs.

### 4.1. Simulation Setup and Datasets

The following sub-section describes the datasets employed to evaluate the proposed algorithm. For a proper assessment, both a scalp EEG dataset and an iEEG dataset were considered. The latter includes short-term sessions, focused on few minutes centered around the ictal activity, and long-term recordings, which provide realistic conditions of extended monitoring periods.

#### 4.1.1. CHB Dataset

For validation, the proposed algorithm was tested on the publicly available CHB-MIT Scalp EEG dataset, collected at Boston Children’s Hospital in collaboration with the Massachusetts Institute of Technology [23]. The dataset contains long-term EEG recordings from 23 pediatric patients with epilepsy, comprising 9 to 40 sessions per subject and totaling over 900 h. Signals were acquired with the international 10–20 electrode system, sampled at 256 Hz with 16-bit resolution, and generally included 23 recording channels. Each recording is accompanied by expert-annotated seizure onset and offset times, providing a benchmark for the statistical analysis of the detector. Information pertaining to the CHB dataset is annotated in Table 2.

#### 4.1.2. SWEC-ETHZ iEEG Database

The long-term SWEC-ETHZ iEEG dataset comprises 2656 h of continuous recordings from 18 patients with pharmaco-resistant epilepsy, collected during pre-surgical evaluation at the Sleep-Wake-Epilepsy-Center (SWEC) of the University Department of Neurology, Inselspital Bern, in collaboration with the Integrated Systems Laboratory of ETH Zurich. The dataset includes 116 annotated seizure events, acquired with strip, grid, and depth electrodes. Signals were median-referenced, band-pass filtered between 0.5 Hz and 120 Hz using a fourth-order Butterworth filter, and digitized at 512 Hz or 1024 Hz with 16-bit resolution [24].

As with the previously mentioned dataset, the recordings are accompanied by annotations that label the beginning and conclusion of seizure events, determined through visual inspection by clinical experts. The overall number of seizures per patient, as well as the average seizure time, have been reported in Table 3. Contrary to the CHB-MIT dataset, it can be observed that the number of channels per patient varies greatly, ranging from 24 to over 100.

To further test the adaptability of the algorithm, the short-term SWEC-ETHZ iEEG dataset was also taken into consideration (Table 4). Such collection of samples includes 100 recordings taken from 16 patients. Each recording contains 3 min of pre-ictal activity, the ictal segment, and 3 min of post-ictal activity [25].

Results from the simulation of the algorithm on the aforementioned datasets are thoroughly analyzed in the coming section.

### 4.2. Performance of the Proposed Algorithm

This section presents the metrics used to calibrate and assess the performance of the proposed algorithm. The subsequent results summarize its application across the selected datasets, with all simulations conducted in the MATLAB environment.

#### 4.2.1. Evaluation Metrics

Before delving into the simulation results, it is necessary to introduce the statistical metrics used to both tune the thresholds and assess the algorithm’s efficiency on test data. First, the definition of accuracy adopted in this work, as defined in [15], is based on the ratio of correctly classified samples to the total number of samples, thus providing a comprehensive performance indicator that accounts for both correct detections and misclassifications. As such, it can be expressed as:

(7) $$\mathrm{Accuracy}=\frac{\mathrm{TP}+\mathrm{TN}}{\mathrm{TP}+\mathrm{TN}+\mathrm{FN}+\mathrm{FP}}\times 100.$$

**Figure 10:**
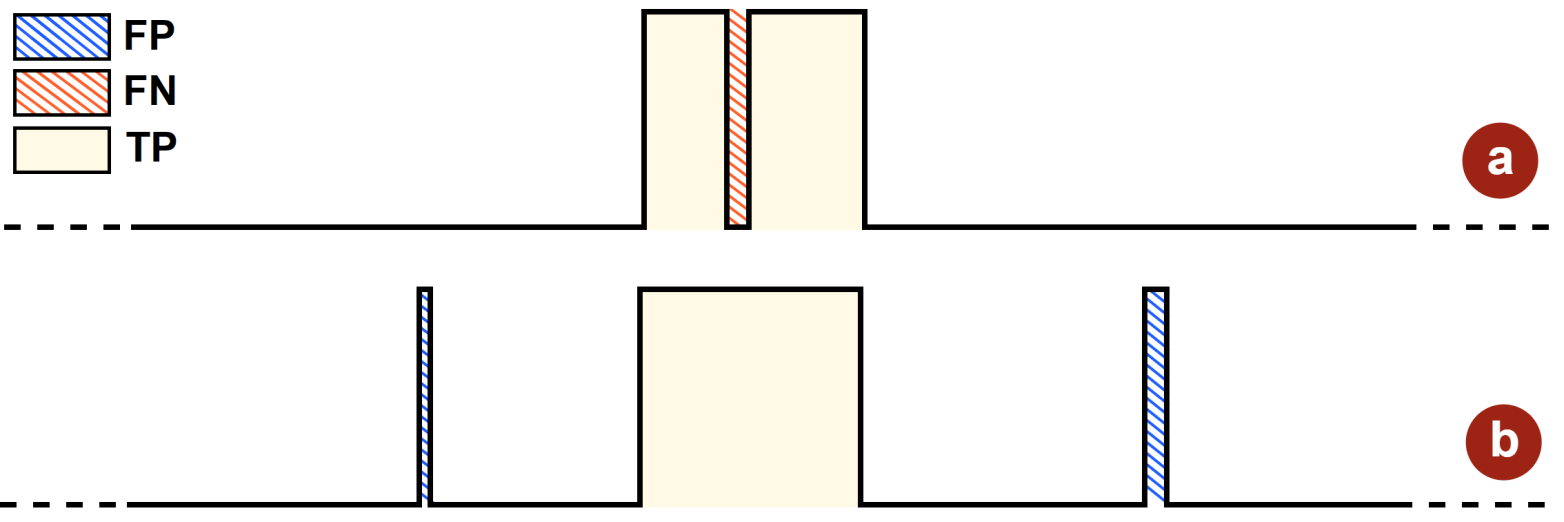
Explanatory illustration of (**a**) false negatives, and (**b**) false positives.

Here, TP denotes the number of true positives, corresponding to correctly detected seizure samples, while FN represents the amount of false negatives, that is, missed detections. FP indicates the portion of false positives, interpreted as incorrect detections during normal neural activity. Finally, TN refers to the number of samples correctly identified as non-seizure related (Figure 10).

In addition, sensitivity is introduced as a metric that quantifies the algorithm’s ability to correctly detect epileptic seizures. It is defined as the ratio between true positives and the sum of true positives and false negatives, thereby measuring the percentage of seizures that are successfully identified. As such, this metric is not influenced by the quantity of true negatives, nor by the number of false positives, as it interests solely the classification of the actual seizure event. The formula for sensitivity is defined as follows:

(8) $$\mathrm{Sensitivity}=\frac{\mathrm{TP}}{\mathrm{TP}+\mathrm{FN}}\times 100.$$

In practice, it depends not only on whether a seizure is detected, but also on how quickly the detection occurs, as delayed detections increase the number of unidentified positives and thus lower the sensitivity.

To quantify the algorithm’s responsiveness, latency has also to be taken into account. The latter refers to the time interval, typically measured in seconds, between the actual onset of a seizure and its detection performed by the algorithm. In this study, where testing is performed on recorded datasets rather than live subjects, latency is calculated as the difference between the detection time and the seizure onset as indicated by the clinical annotation provided with each sample.

The expression for latency adopted throughout the development of this work is therefore equal to:

(9) $$\mathrm{Latency}=T_{onset}-T_{detection},$$

where $T_{onset}$ denotes the timestamp of the seizure’s beginning provided with the test samples, while $T_{detection}$ represents the instant in which the detection first occurs. Specifically, $T_{detection}$ is identified as the earliest time instant within the annotated ictal window from which the algorithm consistently flags seizure-related activity across channels. It must be noted that the metric defined in Equation (9) carries a degree of uncertainty due to the subjective nature of the annotations, with the marked onsets possibly not aligning with the physiological genesis of the epileptic seizures.

**Figure 11:**
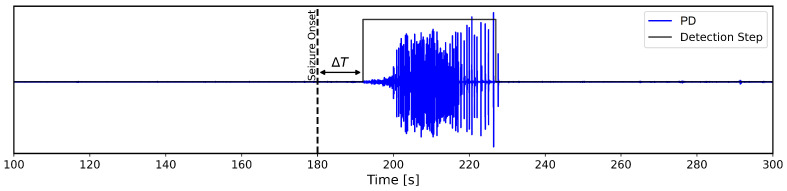
Example case illustrating a detection delay with the threshold-based algorithm, showing the seizure being identified slightly after the annotated onset.

Figure 11 shows a practical example of the delay described above, illustrating how the inherent properties of the threshold-based algorithm can cause its identification of physiological changes in neural patterns to diverge from the annotated seizure onset due to uncertainty in the onset timing.

#### 4.2.2. Simulation Results

Simulations were first conducted on the CHB-MIT dataset, selecting five patients as representative examples. All available seizure samples for these patients were used to evaluate the algorithm. Since each sample is 60 min long, they provided sufficient ictal and inter-ictal data to perform threshold calibration using only the first seizure-containing sample per subject. Such tuning aimed to achieve an optimal trade-off between accuracy and sensitivity. Subsequent test samples were then evaluated using the fixed thresholds obtained from the initial tuning.

The calibration itself was performed individually for each patient in the CHB-MIT dataset and reiterated for those in the other datasets. The process involved iteratively running the algorithm on the sample of data reserved for tuning. Initial thresholds for the *LL* and *PD* features were defined as scaled versions of their standard deviations and progressively adjusted, together with the counter variable $k_{c}$, to maximize detection accuracy. Once satisfactory accuracy was reached, $Thr_{p}$ and $Thr_{LL}$ were gradually lowered to achieve at least 90% sensitivity, or preferably higher, while maintaining accuracy above a target value. Detection latency showed a strong dependence on sensitivity, as higher sensitivity generally reduced missed detections and thus latency. The parameter Λ was contextually optimized based on the number of channels, corresponding to the number of electrodes used to record the neural data, and was set to represent a fixed percentage of the total. The results reported for the CHB dataset, as well as the SWEC-ETHZ datasets presented later, reflect this patient-specific calibration procedure.

For simulations of the algorithm conducted on the CHB dataset, as well as the intracranial SWEC-ETHZ datasets, a duration of 1 s was established for the processing windows, corresponding to $f_{s}$ samples. This configuration was identified as optimal after evaluating multiple window sizes and observing superior overall detection performance and consistency across datasets.

Regarding the scalp EEG CHB-MIT dataset, results reported in Table 5 demonstrate that the proposed algorithm achieves excellent performance across all tested subjects. Average accuracy per patient ranges from 95.95 % (CHB07) to 98.23 % (CHB05), while sensitivity remains consistently high, often approaching 100 %. Detection delays are low, with an overall mean of 3.37 s, and false positive rates are well contained, averaging 2.26 % per hour, supporting the method’s reliability in distinguishing seizure and non-seizure activity. Since the threshold is kept constant across all test samples, there is a slight, though not majorly significant, decrease in accuracy for later seizure events, suggesting potential for improvement through an adaptive thresholding or autocalibration strategy to maintain uniformly high performance over time.

Concerning the short-term SWEC iEEG dataset, threshold calibration required a different approach due to the limited inter-ictal data in each sample. Approximately half of the seizure-marked recordings were used for training to properly tune the thresholds, while the remaining half were reserved for testing. It must be noted that, given the short duration of the samples, particularly in terms of inter-ictal activity, the identification of true positives carries more weight, as there are fewer true negatives available.

The results reported in Table 6 indicate that the proposed algorithm performs very well under these conditions. Accuracy for individual seizures is high, with patient-level averages exceeding 98 % in the examples reported. Sensitivity remains consistently close to 100 %, demonstrating reliable detection of ictal activity. Detection delays are generally low, ranging from 5 to 13 s on average per patient, and false positive rates are minimal, highlighting the method’s reliability even in recordings with limited inter-ictal periods and highly variable electrode configurations.

For the evaluation on the long-term SWEC iEEG dataset, five subjects were selected, similarly to the previous runs on the short-term dataset. As with the CHB dataset, 60 min long recordings were used, and a single sample containing a seizure event was sufficient to tune the algorithm and calibrate the thresholds depending on the tested subject. The results reported in Table 7 show that the algorithm maintains high accuracy and sensitivity, comparable to those obtained on the CHB dataset. However, for both iEEG datasets, the average latency is slightly longer, which can be attributed to a certain degree of uncertainty in the seizure onset annotations in the SWEC recordings. It is also worth noting that some tests show a delay of 0 s or even negative values; despite the algorithm incorporating an intrinsic three-second delay due to the conclusive check over three consecutive windows before marking a seizure, these cases likely reflect the algorithm detecting early pre-ictal physiological fluctuations.

### 4.3. Comparative Analysis with State-of-the-Art

In this section, the results of the simulations conducted on the CHB-MIT and SWEC-ETHZ datasets are compared against other state-of-the-art proposals. Notably, works pertaining to the latter of the two datasets have been considered both regarding the short-form and the long-form variants. As shown in Table 8, comparisons were made with respect to algorithms that were tested on either of the two datasets, with most of them being the product of machine-learning-based approaches. Included in Table 8 are also works that were evaluated on different publicly available datasets, with a distinction being made between EEG and iEEG signals. Additionally, not all references proposed in said table have been implemented.

The reported results show that the proposed work, in spite of its simplicity, manages to yield comparable scores in terms of accuracy and sensitivity, with latency times that do not deviate particularly from the average of about 5 s. The proposed threshold-based algorithm demonstrates high performance on the CHB-MIT dataset, achieving a sensitivity of 98.73% and an accuracy of 97.71% on average. These results are comparable to or exceed those obtained by more complex, machine-learning-based approaches, including 2D-CNNs, SVMs, RUSBoost, and Random Forests, which typically require extensive feature extraction and model training. In addition to its competitive accuracy, the algorithm exhibits a low detection latency of 3.37 s on average, outperforming several ML-based methods that report longer response times. Favorable results were also obtained when evaluating the algorithm on the iEEG dataset against similarly tested approaches. On average across the tested samples, the proposed method achieved a sensitivity of 98.62% and an accuracy of 98.34%, both higher than most existing works and indicative of resilience against false detections. Latency performance was also superior, regardless of the distinction between long-term or short-term variants, yielding an overall average of 7.84 s.

**Figure 12:**
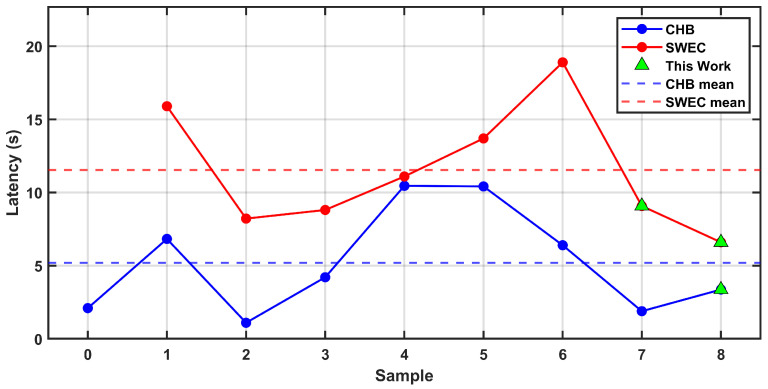
Trends in latency related to detection algorithms applied to CHB or SWEC-EHTZ datasets.

As shown in Figure 12, the latency values reported for various algorithms on the CHB and SWEC datasets reveal notable differences across datasets. For most state-of-the-art methods, latencies increase when tested on the iEEG SWEC dataset compared to CHB, suggesting potential inconsistencies in the annotations or differences in the characteristics of the SWEC recordings. The points corresponding to the proposed approach demonstrate competitive performance on both datasets, achieving low latency comparable to other methods on CHB while maintaining robust performance on SWEC, with results on the long-term SWEC dataset among the best reported. These results suggest that the method generalizes effectively across datasets and further emphasize the importance of cross-dataset validation to account for possible differences in annotation protocols and signal characteristics.

### 4.4. Hardware Resource Comparisons

This section presents the FPGA synthesis results, summarized in Table 9, and juxtaposes them with recent hardware implementations. Here, the strength of this design is particularly highlighted, as the comparisons show that the proposed work relies on minimal resources to be effective. The proposed solution achieves the lowest usage of LUTs and FFs among AMD Artix-7 implementations and requires only 3 DSP blocks, demonstrating an efficient mapping of the algorithm to hardware. Notably, the number of DSPs does not increase with the number of channels, as they are shared via the TDM implementation, unlike with other methods where DSP usage may scale with the channel count. The small overall resource footprint suggests that the algorithm could be implemented on limited platforms or even ASICs to further push the miniaturization process with sub-micron nodes.

## 5. Conclusions

In this work, a low-complexity threshold-based seizure detection algorithm was presented and thoroughly assessed in terms of both performance on pre-existing datasets and hardware resource allocation when implemented on the AMD Artix-7 FPGA board. By exploiting the *PD* and *LL* time-domain features, the architecture is able to isolate and effectively detect the abnormal activity related to the presence of an epileptic seizure, provided that a calibration of the thresholds is carried out beforehand. The accuracy and sensitivity of the proposed system were evaluated using the CHB-MIT scalp EEG dataset and the SWEC-ETHZ iEEG dataset, both in its long-term and short-term variant. After testing on over 35 samples taken from said datasets, the simulated accuracy exceeded on average 98%, with similar results being obtained with regard to the average sensitivity. The latency of the system, when compared against more complex state-of-the-art proposals pertaining to the specific datasets, is among the shortest, with recorded averages of 3.37 s (CHB) and 7.84 s (SWEC).

The simplicity of the algorithm translates into minimal resource requirements for hardware implementation. FPGA synthesis results demonstrated substantial improvements in LUT, DSP, and FF utilization compared with single-channel designs. For fairness, a 4-channel version of the framework was considered in the comparison, ensuring full functionality of the proposed architecture. Moreover, the adoption of a TDM approach for both pre-processing and feature extraction prevents resources usage from scaling linearly with the number of channels, further strengthening the efficiency of the solution. Overall, these results demonstrate that the proposed detection algorithm is accurate, fast, and lightweight, making it well-suited for real-time applications in hardware-constrained environments.

## Figures and Tables

**Table 1:** Synthesis results showing resource utilization on the AMD Artix-7 XC7A100T FPGA with a 4 input channel configuration.

Resources	Utilization	Available	Utilization (%)
LUT	192	63,400	0.30
FF	500	126,800	0.39
DSP	3	240	1.25
BRAM	0.5	135	0.37

**Table 2:** Summary of the CHB dataset, including the number of patients, their age and gender, seizure counts, and average seizure time per patient.

Metric	chb01	chb02	chb03	chb04	chb05	chb06	chb07	chb08	chb09	chb10	chb11	chb12	chb13	chb14	chb15	chb16	chb17	chb18	chb19	chb20	chb21	chb22	chb23
Sex	F	M	F	M	F	F	F	M	F	M	F	F	F	F	M	F	F	F	F	F	F	F	F
Age (years)	11	11	14	22	7	1	14	3	10	3	12	2	3	9	16	7	12	18	19	6	13	9	6
N° Seizures	7	3	7	4	5	10	3	5	4	7	3	35	9	9	21	10	3	6	3	8	4	3	7
Avg. Time (s)	63	57	57	94	112	15	108	183	69	64	268	35	38	19	103	8	98	53	79	37	50	68	61

**Table 3:** Summary of the long-term SWEC-ETHZ iEEG dataset, including the number of channels, seizure counts, and average seizure time per patient.

Metric	ID01	ID02	ID03	ID04	ID05	ID06	ID07	ID08	ID09	ID10	ID11	ID12	ID13	ID14	ID15	ID16	ID17	ID18
N° Ch.	88	66	64	32	128	32	75	61	48	32	32	56	64	24	98	34	60	42
N° Seizures	2	2	4	14	4	8	4	70	27	17	2	9	7	60	2	5	2	5
Avg. Time (s)	602	88	65	42	35	43	53	22	34	42	43	53	50	34	35	43	39	44

**Table 4:** Summary of the short-term SWEC-ETHZ iEEG dataset, including the number of patients, average seizure time, seizure counts, and variability in the number of channels per patient.

Metric	ID01	ID02	ID03	ID04	ID05	ID06	ID07	ID08	ID09	ID10	ID11	ID12	ID13	ID14	ID15	ID16
N° Ch.	47	42	98	62	54	64	36	59	56	100	64	49	92	74	61	59
N° Seizures	13	4	2	14	10	4	2	2	9	5	2	10	7	7	3	6
Avg. Time (s)	71	223	99	153	98	146	15	56	144	14	109	45	81	602	121	88

**Table 5:** Detection metrics per seizure and patient-level averages. CHB Dataset.

Patient	Test Seizure	Accuracy (%)	Sens. (%)	Delay (s)	FPR/h (%)
CHB01	#1	98.91	99.99	0	1.09
	#2	96.90	99.99	0	3.10
	#3	98.36	100	0	1.64
	#4	99.77	100	2	0.23
	#5	97.73	96.47	4	2.18
	#6	96.62	100	3	3.38
	**Avg**	**98.04**	**99.40**	**1.5**	**1.94**
CHB05	#1	99.40	98.05	8	0.55
	#2	99.22	92.39	5	0.57
	#3	99.20	97.41	4	0.72
	#4	95.11	100	13	4.89
	**Avg**	**98.23**	**96.96**	**7.5**	**1.68**
CHB07	#1	96.64	97.77	6	3.30
	#2	95.26	97.65	7	4.66
	**Avg**	**95.95**	**97.71**	**6.5**	**3.98**
CHB09	#1	98.51	99.99	3	1.49
	#2	96.78	99.99	2	3.22
	**Avg**	**97.64**	**99.99**	**2.5**	**2.35**
CHB23	#1	96.93	99.99	2	3.07
	#2	98.00	99.99	−5	2.04
	**Avg**	**97.46**	**99.99**	**−1.5**	**2.55**
**AVERAGE**		**97.71**	**98.73**	**3.37**	**2.26**

**Table 6:** Detection metrics per seizure and patient-level averages. SWEC-ETHZ short-term dataset.

Patient	Test Seizure	Accuracy (%)	Sens. (%)	Delay (s)	FPR (%)
ID2	#1	99.33	100	8	0.67
	#2	97.73	100	3	2.27
	**Avg**	**98.53**	**100**	**5.5**	**1.47**
ID5	#1	96.59	95.83	9	2.73
	#2	99.33	100	13	0.67
	#3	98.48	95.50	14	0.65
	#4	98.51	100	8	1.49
	#5	97.49	100	4	2.51
	**Avg**	**98.08**	**98.27**	**9.6**	**1.61**
ID6	#1	99.38	97.32	14	0
	#2	98.55	98.15	12	0.91
	**Avg**	**98.76**	**97.73**	**13**	**0.45**
ID8	#1	97.39	97.96	14	2.38
ID10	#1	99.73	99.98	4	0.27
	#2	100	99.98	6	0
	**Avg**	**99.86**	**99.98**	**5**	**0.13**
**AVERAGE**		**98.54**	**98.73**	**9.08**	**1.21**

**Table 7:** Detection metrics per seizure and patient-level averages. SWEC-ETHZ long-term dataset.

Patient	Test Seizure	Accuracy (%)	Sens. (%)	Delay (s)	FPR (%)
ID1	#1	99.22	99.29	2	0.67
ID2	#1	97.78	95.18	8	2.11
ID3	#1	99.92	95.16	2	0
	#2	100	100	2	0
	#3	99.92	95.52	3	0
	**Avg**	**99.95**	**96.89**	**2.33**	**0**
ID15	#1	**97.36**	**100**	**13**	**2.64**
ID16	#1	99.64	100	13	0.36
	#2	99.47	100	9	0.53
	#3	99.53	100	7	0.47
	#4	88.61	100	7	11.39
	**Avg**	**96.81**	**100**	**9**	**3.19**
**AVERAGE**		**98.14**	**98.51**	**6.60**	**1.82**

**Table 8:** Comparison of performance metrics against recent state-of-the-art proposals. Colored in yellow are works appied to EEG data, colored in green are works applied to iEEG data.

Reference	Type	%Sens.	%Acc.	Latency (s)	Database
[12]	2D-CNN	100.00	–	2.10	CHB
[16]	RUSBoost	95.00	99.71	6.83	CHB
[13]	SVM	97.20	97.80	1.10	CHB
[17]	ADCD	96.00	–	4.21	CHB
[26]	CNN	97.57	98.90	10.46	CHB
[27]	SVM	96.15	96.38	10.42	CHB
[28]	SVM	95.20	–	6.40	CHB
[29]	Rand. Forest	98.00	98.00	–	UCI-ESR
[30]	RVM	96.00	99.88	1.89	Bonn/CHB
**This Work**	**Thr-Based**	**98.73**	**97.71**	**3.37**	**CHB**
[31]	LBP+HD	96.01	95.42	15.90	SWEC-SH
[14]	Thr-Based	100	–	8.22	SWEC-SH
[25]	LBP	96.38	96.85	8.81	SWEC-SH
[32]	SVM	94.46	–	11.1	Freiburg
[33]	2D-CNN	97.50	–	13.7	SWEC-SH
[24]	Laelaps	85.5	–	18.9	SWEC-LO
**This Work**	**Thr-Based**	**98.73**	**98.54**	**9.08**	**SWEC-SH**
**This Work**	**Thr-Based**	**98.51**	**98.14**	**6.60**	**SWEC-LO**
**This Work**	**Thr-Based**	**98.62**	**98.34**	**7.84**	**SWEC-AV**

SH: short-term; LO: long-term; AV: average results between short-term and long-term.

**Table 9:** Comparison of FPGA synthesis reports.

Reference	Year	Board	LUT	FF	DSP
[34]	2018	M2GL025	5355	357	–
[14]	2022	Cyclone V	1856	3088	5
[35]	2022	Zynq-7000	5202	10,196	–
[36]	2022	Artix-7	5934	4015	–
[19]	2023	Artix-7	1150	492	14
[18]	2025	Altera DE2	1095	–	9
**This Work**	**2025**	**Artix-7**	**192**	**500**	**3**

## Data Availability

The original contributions presented in this study are included in the article. Further inquiries can be directed to the corresponding author.
